# Key aspects of fine-tuning and applying LLM-as-a-judge for clinical data summaries in the radiological workflow

**DOI:** 10.3389/frai.2026.1768005

**Published:** 2026-03-19

**Authors:** Aleksandr Borisov, Tikhon Burtsev, Pavel Kosov, Tatiana Bobrovskaya, Yuri Vasilev, Anton Vladzymyrskyy, Olga Omelyanskaya, Anastasia Pamova, Kirill Arzamasov

**Affiliations:** 1 Research and Practical Clinical Center for Diagnostics and Telemedicine Technologies of the Moscow Health Care Department, Moscow, Russia; 2 Department of Medical Cybernetics and Computer Science, Pirogov Russian National Research Medical University, Moscow, Russia; 3 Department of Information Technology and Medical Data Processing, Federal State I.M. Sechenov First Moscow State Medical University of the Ministry of Health of the Russian Federation (Sechenov University), Moscow, Russia; 4 Department of Artificial Intelligence Technologies, MIREA – Russian Technological University, Moscow, Russia; 5 Department of Radiation Diagnostics and Radiation Therapy, Samara State Medical University, Samara, Russia

**Keywords:** clinical summarization, EHR (electronic health record), large langauge models, LLM-as-a-judge, radiology

## Abstract

**Background:**

This study aims to describe our experience in fine-tuning an LLM-as-a-Judge to evaluate the quality of clinical text summarization in the field of radiology and to formalize the main problems we encountered in solving this task.

**Methods:**

In this study, information from the Russian language electronic medical records of 30 patients was used. Patients who underwent abdominal computed tomography were selected. Anonymized information about complaints, disease history, medical history, and laboratory and instrumental findings was obtained from the electronic medical records of patients. This information was summarized by six large language models. The resulting summarizations were then evaluated by experts and six different LLMs-as-a-Judges. Kendall’s coefficient of concordance was employed to measure consistency.

**Results:**

The primary difficulties that we encountered in the development of LLM-as-a-Judge included the selection of the rating scale, evaluation criteria, various categories of members included in the expert team, and prompt granularity. No definitive association was identified between scale size and the consistency of ratings between radiologist experts and LLMs-as-a-Judges. Across different evaluation criteria, the highest level of consistency was achieved with varying scale sizes. Our results indicate that criteria effective for human text evaluation are not always suitable for assessment via an LLM-as-a-Judge. For the majority of the criteria, the highest consistency was observed when all LLMs-as-a-Judges operated with a detailed description of extreme scale values or without a detailed scale description in the prompt. For the effective development of an LLM judge, it is necessary to involve a diverse team of experts.

**Conclusion:**

For the proper configuration of an LLM-as-a-Judge, numerous factors should be considered, the number of which varies depending on the specific task. To achieve optimal results, additional experiments should be conducted to fine-tune the prompt and other model hyperparameters, comparing their responses against the desired output.

**Clinical trial registration:**

ClinicalTrials.gov, identifier NCT07057830.

## 1 Introduction

Modern trends in healthcare digitalization have led to the development of various artificial intelligence (AI) assistants that automate various routine tasks of doctors and reduce their workload (Vasilev et al., 2025; Vasilev and Vladzymyrskyy, 2025; Ayers et al., 2023). Large language models (LLM) are the basis of many AI assistants. LLMs are effective tools for analyzing large amounts of text data and generating human-like texts and are actively used in healthcare (Thirunavukarasu et al., 2023). LMMs are capable of solving various tasks in healthcare, such as formulating assignments for medical education (Lucas et al., 2024), answering patients’ questions (Yalamanchili et al., 2024), creating discharge epicrises (Patel and Lam, 2023), and supporting medical decision-making (Liu et al., 2023). One of the promising tasks is the generation of automated summaries—extracting and synthesizing key information from many medical records and research results contained in patients’ electronic medical records into a short and comprehensive summary (Tang et al., 2023). LLMs yield good results in summarizing texts, but they often struggle when working with long documents, may miss important information, and remain susceptible to hallucinations (Liu et al., 2024).

In order to choose the most effective LLM for summarizing the medical text, it is necessary to develop a rigorous methodology for evaluating such models, taking into account the specifics of the subject area and the requirements of the model’s end user. The use of expert assessments has always been and remains the gold standard for assessing the quality of LLM’s work. Experts have a holistic mindset, are able to comprehensively evaluate the generated text, and have a deep understanding of the subject area (Tam et al., 2024). However, the expert-based approach is expensive, experts cannot process large amounts of information quickly, and their evaluations are also at risk of potential bias and inconsistency (Wu et al., 2022).

In addition to expert evaluation, automated metrics are actively used: Recall-Oriented Understudy for Gisting Evaluation (ROUGE) measures the overlap between the generated text and the reference text based on the presence of n-grams (sequences of n words) in the texts. Single words (unigrams)—ROUGE-1—or pairs of words (bigrams)—ROUGE-2—are often used (Lin, 2004); Bilingual Evaluation Understudy (BLEU) calculates the number of n-grams in the generated text that match the n-grams in the reference text (Papineni et al., 2002); Metric for Evaluation of Translation with Explicit ORdering (METEOR) calculates the harmonic mean precision and recall of unigrams, taking into account synonyms and stemming (shortening words to their root form) (Banerjee and Lavie, 2005); and Bidirectional Encoder Representations from Transformers Score (BERTScore) leverages contextual embeddings from pretrained BERT and matches words in candidate sentences and reference sentences using cosine similarity (Zhang et al., 2020). These metrics provide good scalability and consistency; however, they primarily assess the coincidence of n-grams at the surface level, without considering the deep semantic interactions of terms and without evaluating the semantic correctness of the transmitted information (Fabbri et al., 2021). Using exclusively automated metrics to evaluate LLMs in medical tasks is complicated since medical texts may contain critical but small semantic details, and these metrics cannot assess the clinical significance, logical coherence, or the semantic accuracy of the facts presented (Croxford et al., 2024).

A promising approach for assessing the quality of LLM summarization is the use of LLMs-as-a-Judges (Gu et al., 2025; Wang et al., 2024). An LLM-as-a-Judge is a separate LLM used to evaluate the results of text generated by other LLMs according to predefined rules, criteria, and preferences. The advantage of using LLMs-as-a-Judges is the possibility of deep customization of model evaluation instructions, along with the possibility of receiving feedback from an LLM-as-a-Judge, explaining why it made a particular decision (Chiang and Lee, 2023). LLM-based judges show good prospects in processing multimodal data (Chen et al., 2024), which are relevant for medical tasks involving various types of data, such as medical examination protocols, radiation examination reports, and laboratory test results. However, at present, information concerning the use of such solutions for medical tasks is limited (Croxford et al., 2025).

The purpose of this study was to describe our experience in fine-tuning an LLM-as-a-Judge to evaluate the quality of clinical text summarization in the field of radiology and to formalize the key challenges we encountered in solving this task.

## 2 Materials and methods

**Figure 1:**
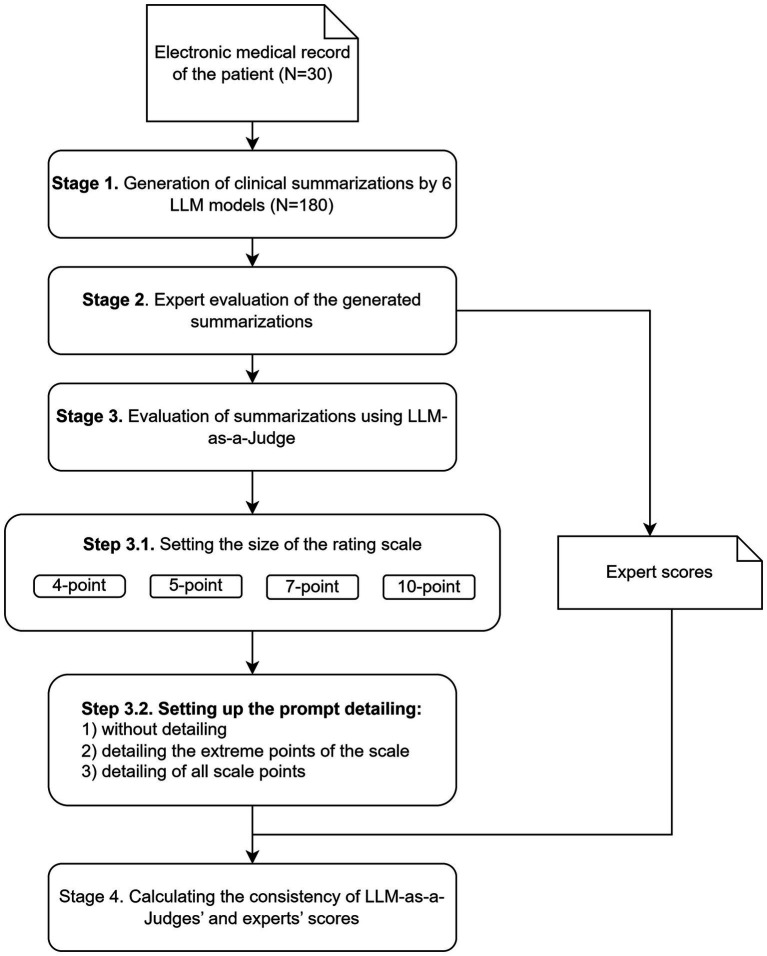
Research workflow.

The study design is presented in Figure 1.

The initial task we addressed was the application of LLMs to generate summaries of information from Russian-language patients’ electronic health records (EHRs) for use by radiologists and to subsequently assess the quality of these summaries.

At the first stage, EHRs were selected and their clinical summaries were generated via LLMs. To obtain the information summarization, the following prompt was provided to the models:

“The patient underwent abdominal computed tomography (CT). The data extracted from the provided text would be useful for a radiologist in describing the abdominal CT according to the following structure:

- Complaints prompt the performance of abdominal CT.
- Disease history.
- Patient’s medical history (comorbidities, harmful habits, family history, and prior surgeries).
- Laboratory data.
- Instrumental examination findings.

Do not include in the response any data unrelated to the task of providing the radiologist with information necessary for describing the abdominal CT.”

Six LLMs were employed for generating the summaries: Qwen3-32B (quantization q4_K_M), Llama-3.3-70B-Instruct (quantization q4_K_M), Gemma-3-12B-it, Llama3.1-8B, Gemma-2-27B-it, and Llama-3.2-3B-Instruct. For text generation, we used the optimal hyperparameters recommended by the developers in the official model card for each model. The source of information for generating the summaries was anonymized data from EHRs of patients referred for abdominal CT. The data were obtained from the Unified Medical Information and Analytical System of Moscow (EMIAS). Patient demographic data, examination findings by the referring physician, protocols of previous instrumental diagnostic studies, laboratory results, and discharge summaries were utilized. A total of 30 clinical cases were generated, resulting in 180 summaries produced by the LLMs. The volume of one EHR ranged from 2,000 to 4,600 Russian words.

At the second stage, the generated summaries were evaluated by experts based on the criteria developed in advance by us, as presented in Table 1.

**Table 1:** Criteria for evaluating LLM summaries.

Criterion	Scale	Explanation
Relevance	1–5	1–The summary is irrelevant to the query; the information is useless. 5–The summary perfectly addresses the query; all key aspects are included, and the information is useful and accurate.
Completeness	1–5	1–The summary omits most clinically significant data. 5–The generated summary includes all clinically significant findings and important details.
Redundancy	1–5	1–The summary contains excessive irrelevant information unrelated to the query. 5–The summary includes only the information necessary to address the task as per the query
Coherence and structure	1–5	1–The summary is unclear and lacks logical flow. 5–The summary is perfectly clear, logically structured, and easy to follow.
Grammar and terminology	1–5	1–The summary contains numerous language and terminology errors, making it unsuitable for use. 5–The summary text fully adheres to linguistic norms and professional terminology, with no errors.
Hallucinations	0–1	0–There is no information in the summary that was not in the original text. 1–The summary contains information that was not in the original text.

Eighteen radiologists with work experience ranging from 1 to 17 years (average 8.5) participated in the expert evaluation of the summary quality of EHRs. Each summary was assessed by a group of three experts, and a consensus decision was reached among them to reduce the subjectivity inherent in individual expert evaluations. In addition, automatic metrics ROUGE-1, ROUGE-2, BLEU, METEOR, and BERTScore were calculated for each summary.

At the third stage, the generated summaries were evaluated via LLMs-as-a-Judges using the same criteria provided to human experts. The models used as LLM-as-a-Judge are shown in Table 2. These models were selected for the experiments because they are publicly accessible (distributed under the Apache 2.0 license), represent some of the most up-to-date versions within their respective model families and class of medium-sized models at the time of the study, and provided a balance between model performance quality and the computational resources required for local deployment. Testing was conducted on a computational configuration utilizing 2 х [NVIDIA RTX 3090] GPUs.

**Table 2:** Characteristics of models used as LLM-as-a-Judge.

Model	Model sizes (Parameters)	Quantization
Granite 4.0 32b-a9b-h (small-h)	32.2B	Q4_K_M
Gpt-oss: 20b	20.9B	MXFP4
T–Pro 2.0	33B	Q4_K_M
Mistral-Small-3.1-24B-Instruct-2503	24B	Q4_K_M
Deepseek-r1: 32b	32.8B	Q4_K_M
Qwen3-32B	32.8B	Q4_K_M

To identify the optimal algorithm for utilizing LLMs-as-a-Judges, the following experiments were carried out:

- Modification of the rating scale size:
- 4-point scale.
- 5-point scale.
- 7-point scale.
- 10-point scale.
- Adjustment of prompt granularity:

◦ Without a detailed scale description.
◦ With a detailed description of extreme scale values.
◦ With a detailed description of all scale points (Table 3).

**Table 3:** Example prompt configurations for varying levels of granularity.

Level of granularity	Example prompt for the “relevance” criterion
Without a detailed scale description	Your task is to evaluate the summary on a scale from 1 to 7 based on the relevance criterion by answering the question: “How well does the LLM’s output align with the request?”
With a detailed description of extreme scale values	Your task is to evaluate the summary on a scale from 1 to 7 based on the relevance criterion by answering the question: “How well does the LLM’s output align with the request?” Rating scale: 1–Extremely low relevance: near-complete absence of key elements 7–Ideal relevance: perfect alignment with the request
With a detailed description of all scale points	Your task is to evaluate the summary on a scale from 1 to 7 based on the relevance criterion by answering the question: “How well does the LLM’s output align with the request?” Rating scale: 1–Extremely low relevance: near-complete absence of key elements 2–Very low relevance: minimal alignment with the request 3–Low relevance: some relevant elements present but significant extraneous content 4–Moderate relevance: main elements present but with omissions 5–High relevance: nearly all key elements included 6–Very high relevance: complete alignment with minimal extraneous content 7–Ideal relevance: perfect alignment with the request

At the fourth stage, the consistency between the responses produced by the LLMs-as-a-Judges and the evaluations provided by human experts was assessed for each configuration variant. Kendall’s coefficient of concordance was employed to measure consistency. For the “Hallucinations” criterion, due to the binary rating scale (0 and 1), concordance was evaluated only for prompts with and without detailing. To normalize the responses of experts and LLMs using different rating scale sizes, the responses were reduced to a single dimension using the Greatest Common Divisor.

## 3 Results

The summarizations obtained by the models were evaluated by experts according to the criteria provided in Table 1, and the standard automatic metrics ROUGE-1, ROUGE-2, BLEU, METEOR, and BERTScore were calculated. Table 4 shows the average results of estimates and metrics, grouped by the models that performed the summarization.

**Table 4:** Average expert estimates and automatic metrics grouped by the models that performed the summation.

Metric	Model					
	Qwen3-32B	Llama-3.3-70B-Instruct	Gemma-3-12B-it	Llama3.1-8B	Gemma-2-27B-it	Llama-3.2-3B-Instruct
Relevance	2.60	3.83	3.27	2.36	3.57	3.80
Completeness	2.57	3.70	3.17	2.30	3.87	3.93
Redundancy	3.17	4.20	3.47	3.17	4.00	3.20
Coherence and structure	3.57	4.43	3.63	3.10	4.17	4.20
Grammar and terminology	4.53	4.70	4.43	3.63	4.73	4.33
Hallucinations	0.70	0.90	0.67	0.60	0.73	0.70
ROUGE-1	0.32	0.38	0.34	0.29	0.37	0.35
ROUGE-2	0.11	0.17	0.14	0.10	0.14	0.17
BLEU	0.08	0.12	0.10	0.06	0.09	0.11
METEOR	0.21	0.28	0.25	0.19	0.27	0.33
BERTScore	0.73	0.77	0.75	0.72	0.76	0.77

The results presented in Table 4 indicate that automated metrics are poorly applicable to our task. Perfect summarization texts for each EHR are not available; therefore, comparisons are only possible with the source text. Metrics based on n-grams—such as ROUGE or BLEU—will typically show reduced results since any summation will be shorter than the original text and will contain fewer words and matching n-grams. METEOR also works with n-grams at the level of phrases and sentences, the number of which will change when summarizing the text. Although BERTScore enables the assessment of the semantic similarity between texts, it does not take into account semantic errors and the adequacy of information presentation; hence, it cannot be used as a benchmark for evaluating medical summaries. In practice, experts may find certain aspects or features of summarization more important, such as the absence of hallucinations or the completeness of the presentation of the material. In addition, the same text may satisfy experts on some criteria and not on others. Therefore, in our experiment, experts rated the summarizations with relatively high scores according to the criterion of Grammar and Terminology (on average 4.4 out of 5) but with substantially lower scores according to the criterion of Completeness (on average 3.26 out of 5). The use of automatic metrics does not allow for such differentiated, criterion-specific evaluation and provides only one general assessment of the text.

Due to these problems, at further stages of the study, we experimented using LLMs-as-a-Judges to evaluate summarizations. During the selection of LLMs-as-a-Judges and the configuration of their usage for our task, we encountered various challenges. In the following sections, we describe four of the most significant issues, in our view.

### 3.1 Selection of the rating scale

The first challenge we encountered in developing criteria for evaluating summary quality pertained to the type and size of the rating scale employed. A common solution for assessing an expert’s stance on a subject is the Likert scale, one of the most widely used formats in surveys (Clark and Watson, 2019). This scale typically presents a series of statements, questions, or criteria, and respondents indicate their degree of agreement, disagreement, or alignment with each statement using a predefined ordinal scale. Responses are structured as an ordered set (e.g., “Strongly Disagree,” “Disagree,” “Neutral,” “Agree,” and “Strongly Agree”). The use of such a scale enables the quantitative assessment of subjective data, facilitating the analysis and interpretation of responses (Sullivan and Artino, 2013).

A key challenge lies in selecting the size of the Likert scale. Increasing the scale size allows for greater response granularity; however, it complicates the expert’s task, increases discrepancies, and reduces distinctions between adjacent scores (Croxford et al., 2025). Consequently, the boundaries between scores such as 5 and 6 or 3 and 4 become ambiguous and poorly interpretable. Additionally, there is an issue related to the parity (even vs. odd) of the number of scale points. Odd-numbered scales are more commonly used and are generally perceived as more intuitive (Joshi et al., 2015). However, they introduce the problem of central tendency, manifested in respondents’ inclination to select the midpoint—a value that is often difficult to interpret meaningfully. Furthermore, a midpoint response may reflect respondent disengagement rather than a genuinely neutral evaluation, and these categories cannot be reliably differentiated (Pornel and Saldaña, 2013).

Within this study, we examined four commonly used scale sizes: 4-point, 5-point, 7-point, and 10-point scales. We subsequently analyzed the evaluations provided by LLMs-as-a-Judges and calculated the consistency of these evaluations against those assigned by human experts across varying scale granularities (Table 5).

**Table 5:** The consistency of experts with the LLM-as-a-Judge’s assessments at different rating scale sizes.

Model	Criterion	Rating scale size			
		4 points	5 points	7 ponts	10 points
Mistral-Small-3.1-24B	Relevance	0.72*	0.76*	0.73*	0.74*
	Completeness	0.62*	0.73*	0.72*	0.71*
	Redundancy	0.59*	0.57	0.54	0.64*
	Coherence and structure	0.61*	0.63*	0.62*	0.57
	Grammar and terminology	0.58	0.60*	0.59*	0.62*
	Consistency in general	0.62*	0.66*	0.64*	0.66*
Qwen3-32B-Q4	Relevance	0.70*	0.71*	0.67*	0.70*
	Completeness	0.55	0.70*	0.63*	0.69*
	Redundancy	0.59*	0.59*	0.59*	0.63*
	Coherence and structure	0.51	0.58	0.50	0.61*
	Grammar and terminology	0.54	0.56	**0.58**	**0.58**
	Consistency in general	0.58	0.63*	0.59*	0.64*
gpt-oss_20b	Relevance	0.70*	0.75*	0.70*	0.69*
	Completeness	0.66*	0.67*	0.69*	0.67*
	Redundancy	0.56	0.56	0.58	0.62*
	Coherence and structure	0.55	**0.58**	0.56	0.53
	Grammar and terminology	0.61*	0.61*	0.57	0.57
	Consistency in general	0.62*	0.63*	0.62*	0.62*
Granite 4.0 32b-a9b-h	Relevance	0.51	0.61*	0.58	0.53
	Completeness	0.50	0.61*	0.63*	0.58
	Redundancy	**0.46**	0.45	0.42	0.41
	Coherence and structure	0.47	0.59*	0.53	0.53
	Grammar and terminology	0.49	**0.56**	0.45	0.45
	Consistency in general	0.49	**0.56**	0.52	0.50
deepseek-r1_32b	Relevance	0.62*	0.62*	0.61*	0.63*
	Completeness	0.54	0.64*	0.61*	0.62*
	Redundancy	0.59*	0.56	0.52	0.59*
	Coherence and structure	0.52	**0.55**	0.51	0.50
	Grammar and terminology	0.55	0.59*	0.47	0.59*
	Consistency in general	0.56	0.59*	0.54	0.59*
T–Pro 2.0	Relevance	0.70*	0.72*	0.72*	0.73*
	Completeness	0.68*	0.70*	0.69*	0.66*
	Redundancy	0.62*	0.64*	0.62*	0.56
	Coherence and structure	0.55	0.58	0.60*	0.60*
	Grammar and terminology	0.59*	0.60*	0.59*	0.54
	Consistency in general	0.63*	0.65*	0.64*	0.62*

^*^Kendall’s W score is statistically significant (*p* > 0.05).The maximum consistency values for each criterion of the evaluated model are highlighted in bold.

When reviewing the responses of LLMs-as-a-Judges, periodic inconsistencies in scores assigned to the same summary across different scale granularities became apparent. For example, the LLM-as-a-Judge Mistral evaluated the completeness of Summary №10 as follows: 3 out of 4 points, 4 out of 5 points, 6 out of 7 points, and 4 out of 10 points. It is evident that, during the first three evaluations, the model highly rated the summary’s completeness, awarding nearly maximum scores, whereas under the 10-point scale, it rated the same text below average for the same criterion. Notably, when explaining its decision, the model identified identical textual deficiencies across all four evaluations; however, in one instance, it attributed greater significance to these shortcomings. The LLM-as-a-Judge Qwen similarly exhibited this issue, with occasional individual scores deviating from the model’s overall assessment of a specific text. This phenomenon occurs across all scale sizes but is more pronounced at larger scales (7-point and 10-point scales).

We attribute this phenomenon to the interpretation of numerical values by the large language model. The model may conceptualize numerical values differently from human experts, and the larger the scale size is, the greater the potential for response variation. Consequently, inconsistent evaluations occur more frequently with larger scale sizes. Model fine-tuning could mitigate this issue; however, this requires high-quality training data that clearly delineate the distinctions between adjacent scores. Compiling such datasets represents a complex and non-trivial task.

No definitive influence of scale size on the consistency between radiologist-experts and LLMs-as-a-Judges was identified. For different evaluation criteria, the highest consistency was achieved with varying scale sizes. However, it is worth noting that, for the criteria “Relevance” and “Completeness,” most LLM judges achieved the best consistency on a 5-point scale. Overall consistency was highest with a 5-point scale and a 10-point scale for all models. All models tended to have the highest consistency, from moderate to high agreement according to the criteria of “Relevance” and “completeness,” and lower according to other criteria, indicating a greater potential for using these two criteria, especially when involving LLM judges for evaluation. The Mistral-Small-3.1-24B model showed the best consistency indicators according to these criteria.

An interesting pattern can be observed using the example of the T–Pro 2.0 model. This model is a modification of the Qwen3-32B model, which was further trained on Russian-language texts. In our experiment, the consistency of the T–Pro 2.0 model with experts was higher by all criteria and on all scales than the original Qwen3-32B model. This demonstrates the great promise of using LLMs-as-a-Judges models that have been further trained in local languages compared to the basic models.

### 3.2 Selection of evaluation criteria

Most existing evaluation scales focus on assessing the quality of human-written texts and do not encompass all the elements necessary for evaluating summaries generated by LLMs (Stetson et al., 2012). Additionally, the objectives of a questionnaire designed for an LLM-as-a-Judge differ from those intended for a human expert. The expert’s goal is to assess text quality comprehensively, so the questionnaire should assist them in performing this evaluation. In contrast, the primary objective of a questionnaire for an LLM-as-a-Judge is to evaluate the judge’s ability to assess model outputs, followed by evaluating text quality according to criteria that the LLM-as-a-Judge can reliably assess. These differing objectives necessitate distinct approaches to designing such questionnaires.

While developing the evaluation criteria for our LLM-as-a-Judge, we encountered the issue that many criteria useful for human evaluation are either inapplicable or poorly applicable when used by an LLM-as-a-Judge. For example, in our initial study design, experts employed the “Satisfaction” criterion to evaluate LLM-generated summaries, reflecting the degree to which the expert was satisfied with the overall summary. However, this criterion is highly subjective and difficult to formalize, making it challenging to explain how LLM-as-a-Judge interprets it correctly. A similar issue arises with other weakly formalizable criteria. Consequently, the number of criteria used for evaluation via LLMs-as-a-Judges was reduced to the list presented in the “Materials and Methods” section.

We concluded that questionnaires designed for testing LLMs-as-a-Judges should differ from those intended for human completion. All criteria provided to an LLM-as-a-Judge must be unambiguously interpretable, and subjective criteria should be avoided. It is also advisable to clarify for the model the type of response expected for each criterion and the underlying meaning intended. Furthermore, merely providing the judge with the name and interpretation of a criterion is often insufficient; it is necessary to evaluate the model’s response and refine the prompt iteratively until the model learns to assess the text as intended. This process may require multiple iterations.

The validation of questionnaires for humans has been extensively described in the scientific literature and relies on the fact that individuals tend to forget their previous responses over time, necessitating retesting procedures (Vasilev et al., 2024). Such an approach is irrelevant for LLMs, as models with identical configurations will almost invariably produce the same response to the same question. Therefore, questionnaire validation for LLMs should focus on aligning the model’s response with the desired outcome for the given query. If the model’s response does not meet the expectations, additional clarifying information should be provided, and the evaluation criteria should be refined accordingly.

### 3.3 Selection of the expert team

The experience and qualifications of an expert can significantly influence how they interpret and evaluate generated text. To a large extent, evaluations are shaped by the personal interpretations and beliefs of the evaluators regarding the task. The majority of experts may mitigate the influence of these factors; however, time and financial constraints limit the number of feasible human evaluations (Croxford et al., 2025). In medical tasks, expert subjectivity plays a decisive role. Medicine is a domain that is difficult to formalize, where decision-making largely depends on understanding the context of the task, the specialist’s clinical experience, education, worldview, and established approaches and guidelines within the medical community (Ten Cate and Regehr, 2019; Virk et al., 2020). Evaluations in medical studies are often non-objective, vary significantly among experts, and are summarized through achieving a consensus constrained by certain assumptions (Newble et al., 1980). These characteristics undoubtedly complicate the processes of evaluating LLM effectiveness in medicine.

In our study, the evaluators were radiologists who would, in practice, work with LLM-generated summaries. However, even at the task formulation stage, we encountered disagreements among the experts. The experts had varying expectations regarding the use of summaries and imposed different requirements on them. These requirements were associated with the clinical profile in which the physician operates. For example, a radiologist primarily working with oncology patients expects the summary to include information on the types of therapy administered to the patient, the number of therapy cycles, and oncological markers in laboratory tests. Conversely, such information would be unnecessary for a radiologist interpreting preventive screenings, who would deem it redundant. Physicians working in emergency or pediatric settings have their own specific requirements. Beyond the organizational profile, the imaging modalities, predominantly interpreted by the radiologist, may also influence their requirements. For example, volumetric imaging studies, such as computed tomography (CT) or magnetic resonance imaging (MRI), may require more supplementary information compared to planar imaging studies. The radiographic school and approaches established within the medical institution where the physician works may also shape their expectations from the LLM-generated summary. For example, some experts deemed it necessary to include information about medications taken by the patient, while others considered this information superfluous when interpreting the study.

To develop a model that satisfies the needs of the majority of users, particular attention must be given to assembling the expert team. The team should ideally include the largest possible number of representatives from the end-user groups of the model. Our study included physicians from various medical facilities, different subspecialties, and varying levels of work experience. To facilitate interaction among such a large group of experts, the involvement of a cognitive scientist is necessary to establish appropriate communication with the experts and prevent excessive disputes among them (Hoffman, 1987).

### 3.4 Prompt granularity

Various approaches exist for presenting evaluation criteria to an LLM-as-a-Judge. The simplest method involves requesting a score within a specified range, for instance, “Evaluate the relevance of this text on a scale from 1 to 5.” A second approach entails providing a detailed description of the extreme values of the rating scale. For example, “Evaluate the relevance of this text on a scale from 1 to 5, where 1 indicates that the summary is entirely irrelevant to the request, and 5 signifies that the summary perfectly aligns with the request, encompassing all key aspects.” While this approach should theoretically assist the model in correctly understanding the task, it requires precise formulation by the developer. A third, even more complex, approach involves providing a detailed breakdown of the criteria for assigning each score on the rating scale (Bai et al., 2023). Within our study, all three approaches were tested, and the consistency between model assessments and those of physician-experts was evaluated. The results are presented in Table 6.

**Table 6:** Consistency between expert evaluations and LLM-as-a-Judge assessments across varying prompt granularity.

Model	Criterion	Level of granularity		
		Without detailed scale description	With detailed description of extreme scale values	With detailed description of all scale points
Mistral-Small-3.1-24B	Relevance	0.75*	0.76*	0.71*
	Completeness	0.73*	0.73*	0.71*
	Redundancy	0.56*	0.57*	0.56*
	Coherence and structure	0.60*	0.63*	0.56
	Grammar and terminology	0.60*	0.60*	0.57
	Consistency in general	0.65*	0.66*	0.62*
Qwen3-32B-Q4	Relevance	0.70*	0.71*	0.63*
	Completeness	0.65*	0.70*	0.57
	Redundancy	0.60*	0.59*	0.57
	Coherence and structure	0.56	**0.58**	0.57
	Grammar and terminology	0.62*	0.56	0.63*
	Consistency in general	0.63*	0.63*	0.59*
gpt-oss_20b	Relevance	0.73*	0.75*	0.72*
	Completeness	0.66*	0.67*	0.71*
	Redundancy	0.48	**0.56**	0.55
	Coherence and structure	0.59*	0.58	0.55
	Grammar and terminology	0.62*	0.61*	0.59*
	Consistency in general	0.62*	0.63*	0.62*
Granite 4.0 32b-a9b-h	Relevance	0.59*	0.61*	0.58
	Completeness	0.61*	0.61*	0.56
	Redundancy	0.43	**0.45**	0.44
	Coherence and structure	0.59*	0.59*	0.57
	Grammar and terminology	**0.57**	0.56	0.54
	Consistency in general	**0.56**	**0.56**	0.54
deepseek-r1_32b	Relevance	0.61*	0.62*	0.57
	Completeness	0.64*	0.64*	0.60*
	Redundancy	0.50	**0.56**	0.53
	Coherence and structure	**0.57**	0.55	0.44
	Grammar and terminology	0.57	0.59*	0.58
	Consistency in general	0.58	0.59*	0.54
T–Pro 2.0	Relevance	0.70*	0.72*	0.72*
	Completeness	0.67*	0.70*	0.65*
	Redundancy	0.62*	0.64*	0.59*
	Coherence and structure	0.62*	0.58	0.62*
	Grammar and terminology	0.58	0.60*	0.57
	Consistency in general	0.64*	0.65*	0.63*

^*^Kendall’s W score is statistically significant (*p* > 0.05).The maximum consistency values for each criterion of the evaluated model are highlighted in bold.

For the majority of the criteria, the highest consistency was observed when all LLMs-as-a-Judges operated with a detailed description of extreme scale values or without a detailed scale description in the prompt. A configuration with a detailed description of the extreme values of the scale ensures maximum or equal maximum consistency for all six models. For four models—Mistral-Small-3.1-24B (W = 0.66), gpt-oss_20b (W = 0.63), deepseek-r1_32b (W = 0.59), and T-Pro 2.0 (W = 0.65)—this configuration provides a strictly higher consistency compared with the minimum scale description. For the two remaining models—Qwen3-32B-Q4 and Granite 4.0 32b—both less detailed configurations—both the minimum description and the detailed description of the extreme values—provide identical consistency (W = 0.63 and W = 0.56, respectively). It is fundamentally important that the configuration with a detailed description of all intermediate points of the scale is not optimal for any of the studied models; moreover, for a number of models (in particular, deepseek-r1_32b and Granite 4.0 32b), it leads to a noticeable decrease in consistency. Although increased granularity was expected to simplify the task for the LLM, it appears that extensive textual descriptions tend to confuse the model, leading to less consistent evaluations. In a significant proportion of cases, the LLM-as-a-Judge assigned three different scores to the same text across the three prompt granularity variants. Additionally, a tendency toward score underestimation was observed when the most detailed prompt configuration was employed. This phenomenon can be explained by the model attempting to simultaneously account for numerous details; upon failing to identify one or more elements, it lowers the overall score despite the text largely conveying the required information accurately.

We consider the most optimal scenario for our task to involve detailed descriptions of extreme scale values for most criteria and minimal prompt granularity for certain criteria. This approach enables moderate-to-high consistency with expert evaluations to be achieved. The criteria with the highest consistency, as in the experiment with the selection of the scale size, were Relevance and Completeness. The Mistral-Small-3.1-24B model showed the best consistency indicators according to these criteria, as in the experiment with the selection of the scale size. This indicates the high prospects of using this model as an LLM-as-a-Judge for this task.

We also assessed the effect of prompt granularity on LLM-as-a-Judge’s ability to detect gross hallucinations in the text. In this task, hallucination indicated the presence of information in summary that was not contained in the source text, or a free interpretation of information in the source text that did not correspond to reality. Since a binary scale was used to assess the presence of hallucinations, we evaluated only two levels of prompt granularity (without detailed scale description and with detailed description of all scale points). The results are presented in Table 7.

**Table 7:** Consistency between expert evaluations and LLM-as-a-Judge assessments in detecting hallucinations across varying prompt granularity.

Criteria	Model	Level of granularity	
		Without detailed scale description	With detailed description of all scale points
Hallucinations	Mistral-Small-3.1-24B	0.38	**0.41**
	Qwen3-32B-Q4	**0.52**	0.42
	gpt-oss_20b	0.37	**0.38**
	Granite 4.0 32b-a9b-h	0.44	**0.55**
	deepseek-r1_32b	**0.48**	0.44
	T–Pro 2.0	**0.47**	0.42

The maximum consistency values for each criterion of the evaluated model are highlighted in bold.

There was no definitive influence of prompt granularity on the consistency between radiologist experts and LLMs-as-a-Judges in detecting hallucinations. An interesting feature is that all of the models based on the Qwen architecture in our experiment (Qwen3-32B-Q4, deepseek-r1_32b, T–Pro 2.0) showed higher consistency in the absence of scale detail, while other models increased their consistency when explanations were added. In general, all models showed rather low consistency with experts, which indicates the weak ability of LLM to detect semantic hallucinations in medical texts.

At the same time, LLM-as-a-Judge could both skip hallucinations generated by other LLMs and signal the presence of hallucinations that do not exist. Examples of such cases are presented in Table 8. In some cases, models come up with non-existent information or generate similar but missing information based on context. LLM-as-a-Judge is not always able to identify such events. Both summarizing LLMs and LLM-as-a-Judge have a problem analyzing numbers and dates, which complicates the correct interpretation of the chronology of events and can lead to information distortion. Our results indicate that it is not advisable to use LLM-as-a-Judges to search for hallucinations in texts, especially when analyzing a semantically difficult medical text.

**Table 8:** Examples of LLM-as-a-Judge work with hallucinations.

A fragment of the EHR text	A fragment of the LLM summarization	LLM-as-a-Judge assessment	The presence of a hallucination
Anamnesis vitae He has been denying contact with infectious patients for the last 4 weeks. Chronic diseases: chronic bronchitis. Smokes for more than 30 years, 4 packs a day	Anamnesis vitae: Smokes for more than 30 years, 4 packs a day. He abuses alcohol.	There is no information missing from the original text in the summarization.	Information about alcohol abuse is missing from the source text
Complaints Abdominal pain, diffuse, pronounced, worse after eating, without relief after defecation, nausea, and general weakness.	The patient was admitted to the hospital with complaints of shortness of breath, cough with sputum, and fever.	There is no mention in the original text that the patient was admitted with complaints of cough with sputum and fever.	The model did indeed indicate incorrect patient complaints. The complaints were made up based on medical history (the patient had Covid-19 last year)
The main diagnosis ICD-10 code: C34.3–Malignant neoplasm of the lower lobe, bronchi or lung Detailed clinical diagnosis: squamous cell carcinoma of the right lung T4N3M1, IVa. Mts in the brain.	Diagnosis: Metastatic lung cancer (NSCLC) with damage to the right lung.	The mention that the patient has a diagnosis of NSCLC is not confirmed by the source text, since the source text indicates the diagnosis of C34.3–Malignant neoplasm of the lower lobe, bronchi, or lung.	Squamous cell carcinoma belongs to the NSCLC group, so there is no LLM error. However, this is an interpretation of information, which is not always accurate.
Chest X-ray from 4 April 2025 Pulmonary fields: Focal and infiltrative changes were not detected. Vascular pattern: moderately deformed, with signs of moderate congestion in the small circle of blood circulation …	Chest X-ray (4 April, 2025): Focal and infiltrative changes were not detected. The pulmonary pattern is moderately deformed, with signs of moderate pulmonary congestion…	The summarization mentions “Chest X-ray (4 April, 2025)” with a detailed description of its results; however, radiography April 4, 2025 is not presented in the source text. Instead, the source text contains information about radiography on 05 May, 2021	The source text contains information about radiography from 4 April, 2025. LLM-as-a-Judge made a mistake

### 3.5 Limitations of the study

The limitation of our study is the small sample size of the source texts of the EHR. An increase in the number of initial EHRs may increase the objectivity of the conclusions, but it will complicate the expert and automatic evaluation of the obtained summarizations, since 1 EHR in this experiment receives 108 expert and 1,368 LLM-as-a-Judge primary estimates.

Moreover, in this study, a limited number of LLMs were used both to generate summarizations and to evaluate them. The field of language models is constantly evolving, with new models or their configurations appearing almost daily, which could potentially be more effective for the task being solved. We chose the models that, in our opinion, were optimal for our capabilities at the time of the study, but we could have missed a potentially effective candidate for evaluation.

## 4 Conclusion

The use of LLMs as judges represents a promising method for the automated evaluation of texts generated by other LLMs. Although this approach does not yield perfect consistency with human evaluations, it becomes essential when processing large volumes of data that cannot feasibly be assessed solely through human effort. This approach is particularly relevant for medical tasks, where expert time is highly valuable, and it would be impractical to utilize it for reviewing extensive quantities of generated texts during neural network model development.

For proper configuration of an LLM-as-a-Judge, numerous factors should be considered, the number of which varies depending on the specific task. Importantly, criteria effective for human text evaluation are not always suitable for assessment via an LLM-as-a-Judge. To compile the most relevant list of evaluation criteria, a team of medical experts should be engaged. Ideally, these experts should represent diverse end-user groups of the developed solution, enabling the identification of necessary compliance criteria from multiple perspectives. To achieve optimal results, additional experiments should be conducted to fine-tune the prompt and other model hyperparameters, comparing their responses against the desired output. For each specific task and set of evaluation criteria, such configurations may differ and involve various combinations.

## Data Availability

The datasets presented in this article are not readily available because the possibility of storing sensitive personal information about the patient in EHR texts and summaries, but are partly available from the corresponding author upon reasonable request. Requests to access the datasets should be directed to AB, aleksandrborisov10650@gmail.com.
